# Predictors of Mothers’ Care Seeking Behavior for Common Childhood Illnesses: Findings From the Afghanistan Health Survey 2015

**DOI:** 10.34172/ijhpm.2023.7598

**Published:** 2023-11-26

**Authors:** Essa Tawfiq, Khwaja Mir Islam Saeed, Sayed Ali Shah Alawi, Jammalluddin Jawaid, Syed Nasir Hashimi

**Affiliations:** ^1^The Kirby Institute, University of New South Wales, Sydney, NSW, Australia; ^2^Global Health Development/Eastern Mediterranean Public Health Network (GHD/ EMPHNET), Amman, Jordan; ^3^Afghanistan Field Epidemiology Training Program, Afghanistan National Public Health Institute, Kabul, Afghanistan; ^4^Afghanistan Pediatric Association, Kabul, Afghanistan; ^5^Agency for Assistance and Development of Afghanistan, Kabul, Afghanistan; ^6^Jhpiego, Kabul, Afghanistan

**Keywords:** Care Seeking Behaviour, Childhood Illness, Afghanistan

## Abstract

**Background:** Mothers’ care seeking behavior for childhood illness is a key factor of utilizing healthcare for children. We examined predictors of mothers’ care seeking for common childhood illnesses.

**Methods:** This was a cross-sectional study, using data from the Afghanistan Health Survey (AHS) 2015. Data were used from women who sought healthcare for their unwell children. The women were asked whether their children were sick with fever, cough, faster breathing, or diarrhea in the past 2 weeks. The outcome variable was defined as whether the mother sought healthcare for her unwell child from a public clinic, a private clinic, or from a pharmacy store. The Andersen’s healthcare seeking behavior model was used and multinomial regression analysis applied.

**Results:** There were 4979 women, aged 15-49 years, whose under-5 children were sick in the past 2 weeks. Thirty-nine percent of women sought healthcare for their children from a health provider. Mother’s age, child’s age, child’s sex, socioeconomic status, mothers’ perceived severity of childhood illness, and number of under-5 children were predictors of mothers’ care seeking behavior. The likelihood of healthcare seeking was lower for older children (Adjusted odds ratio [OR] [95% CI]: 0.51 [0.37-0.70] from public clinics; 0.33 [0.23-0.47] from private clinics; 0.36 [0.22-0.61] from pharmacy stores), and for girls (Adjusted OR [95% CI]: 0.74 [0.59-0.93] from private clinics). The likelihood of healthcare seeking was greater for children whose mothers knew symptoms of childhood illness (Adjusted OR [95% CI]: 2.97 [1.44-6.16] from public clinics; 7.20 [3.04-17.04] from pharmacy stores). The likelihood of healthcare seeking for children was greater in older mothers (Adjusted OR [95% CI]: 1.54 [1.11-2.12]). It was less likely for the mothers who have more children to seek healthcare for their children (Adjusted OR [95% CI]: 0.53 [0.43-0.65] from public clinics; 0.61 [0.48- 0.79] from private clinics; 0.51 [0.37-0.69] from pharmacy stores).

**Conclusion:** Health policy-makers may opt to use our findings, particularly mothers’ knowledge (perceived severity) of symptoms of childhood illness to develop interventions to enhance timely assessment and effective treatment of common preventable childhood illnesses.

## Background

**Key Messages**

**Implications for policy makers**

- There is a lot of room to improve healthcare seeking for childhood illness, as our findings showed that healthcare was sought for only 2 out of 5 unwell under-5 children. Considering the Afghanistan context, the United Nations (UN) agencies and international donors in the health sector may opt to discuss with the Ministry of Health to explore and adapt culturally appropriate health education interventions, with focus on common childhood illnesses, at the primary care level.
- Substantial gender inequity exists in the utilization of health services for under-5 children. Policy interventions aimed at improving gender equity in utilization of health services for children are desirable.
- Mothers’ knowledge of symptoms of childhood illness, which measures perceived severity of childhood illness in our study, is crucial to reduce childhood illnesses and deaths. Our findings provided evidence that the more a mother knows the symptoms of childhood illness the greater would be the likelihood that the mother seeks healthcare for her child.
- Policy-makers may opt to use our findings and develop policies and strategies to improve mothers’ care seeking behavior for unwell children, particularly for girls.

**Implications for the public**

The likelihood of healthcare seeking for younger children is higher than older children. This can be considered a good practice because younger children get more serious illness and need more attention by their parents. On the other hand, our study shows that when a mother has more children, it is less likely she seeks healthcare for her unwell children in comparison to a mother who has fewer children. Furthermore, our study shows that seeking healthcare from private clinics for girls compared to boys is less likely. This may suggest a preferential attitude of parents towards their male children. Parents should pay equal attention to their children and take them to a health provider when the children are sick, regardless of the child’s sex and how many children live in the household. Parents, particularly mothers are encouraged to learn more about symptoms of common childhood illness through participating in health education sessions at health facilities or any other platforms suitable in the society.

Child death rates are unacceptably high in low- and middle-income countries (LMICs), and poor healthcare seeking behavior for preventable childhood illnesses is a key obstacle to increasing utilization of available healthcare and reducing morbidity and mortality among children under 5 years of age (under-5 children). Globally, in 2020 five million children died before reaching their fifth birthday.^1^ Childhood illnesses such as fever, diarrhea, and acute respiratory infection pose serious health issues in children in LMICs^2^; acute respiratory infection, diarrhea and/or fever are the cause of death in 3 out of 4 under-5 children who die in LMICs.^2^ In Afghanistan, despite the gradual decline in under-5 mortality rate from 178 deaths per 1000 live births in 1990, to 129 in 2000 and to 58 deaths per 1000 live births in 2020,^1^ it is anticipated that the under-5 mortality would rise due to the disruption of and reduction in the amount of donor funding to the Afghanistan health system since the takeover of political power by the Taliban in August 2021.^3,4^ The pressure on the health system to collapse has been compounded by the ongoing complex humanitarian crises and declining economic status of the Afghan society,^5,6^ which contributed to the growing concern on increasing poverty, hunger, unemployment, migration in the population, and malnutrition, diseases, and deaths in children. The United Nations (UN) and partners launched funding appeals for Afghanistan at several international platforms, and the international donors stepped up to keep Afghanistan from a worsening humanitarian crisis by saving the health system from collapse. With pledges from the donors to continue funding the health system, it is likely that the health system would be able to provide basic healthcare to Afghan residents, even if the funding amounts would not be as much as the UN and partners appealed for Afghanistan.^7^

 The Integrated Management of Childhood Illness (IMCI) strategy has been implemented in LMICs since late 1990s.^8-10^ In Afghanistan, significant improvement has been achieved over the past two decades,^11^ which may have contributed to the implementation of IMCI strategy and subsequent improvement in child health and child well-being.^12^ IMCI strategy aims to prevent deaths and diseases while improving the quality of care for under-5 children. It consists of three parts: (*i*) Improving the skills of healthcare workers by providing training and guidelines, (*ii*) Improving how healthcare systems are organized and managed, including access to supplies, and (*iii*) Visiting homes and communities to promote good child rearing practices and good nutrition, while encouraging parents to seek healthcare for their children when the children get sick.

 Improving healthcare seeking behavior of mothers can contribute to the timely assessment and effective treatment of common causes of childhood illness, and reducing deaths among under-5 children in LMICs.^13^ However, considerable number of mothers do not take their unwell children to health facilities,^14^ due to a number of factors, including household income, mother’s illiteracy, mother’s age, type of support mothers receive from their husbands, financial hardship, and distance to health facilities.^14-16^ Sociocultural factors of parents play a significant role in the decision to seek healthcare for their children’s illnesses.^16-19^

 In LMICs, research shows that mothers usually have little knowledge of common symptoms of childhood illness.^16,17^ Research shows that mothers seek healthcare from traditional healers before opting for health facilities for their children’s illnesses.^16,17,19^ Several studies reported that characteristics such as child’s age, child’s sex, mother’s education level, distance to a health facility, residential area, and household socioeconomic status are key influential factors of healthcare seeking practices for childhood illness.^17-26^

 Timely healthcare seeking by the parents may prevent morbidity and mortality in under-5 children,^16,27^ and yet improving healthcare seeking behavior remains a challenge today.^28^ Delays in seeking healthcare by mothers to take their children to a health facility may be associated with higher child mortality.^29^ Delays in seeking healthcare by a mother can lead to the worsening of a child illness which makes the medical care provided to the child to be ineffective. Timely healthcare seeking behavior of parents for their children is a crucial factor in preventing child morbidity.^30^ In Afghanistan, influential factors associated with healthcare seeking behavior of mothers for their children have remained insufficiently studied.

 The aim of this study was to examine predictors of mothers’ care seeking behavior for common childhood illnesses. The findings from this study may have the potential to influence healthcare policies and interventions to encourage timely healthcare seeking behavior and improve diagnoses and effective treatments of childhood illness.

## Methods

###  Study Design

**Figure 1:**
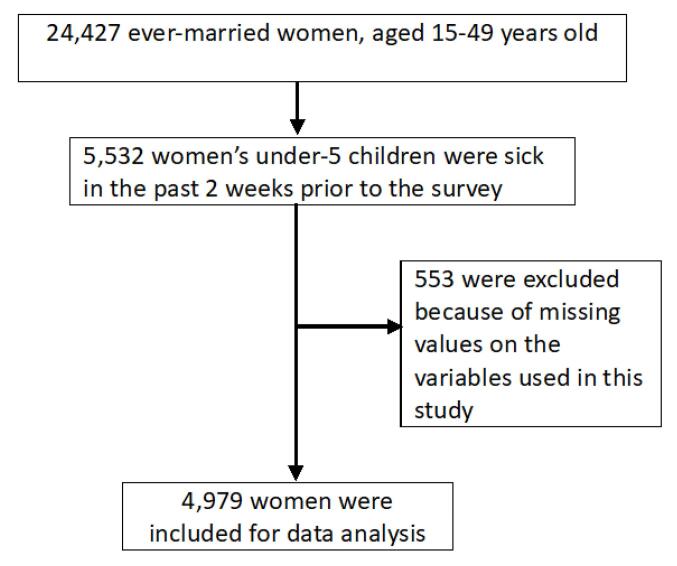
Number of Mothers Entered in the Study.

 The study used secondary data from the Afghanistan Health Survey 2015 (AHS 2015). This was a cross-sectional study, using data collected from households for the AHS 2015.^31^ Data were collected between July and December 2015 from 23 137 households across all 34 provinces of Afghanistan. In this study we included ever-married women, aged 15-49, who had under-5 children. As part of the AHS 2015 questionnaires, women were asked whether they sought healthcare from a health provider for their under-5 children who were sick because of fever, cough, diarrhea, or faster breathing in the past 2 weeks prior to the survey. A total of 24 427 ever-married women, aged 15-49 years, who had under-5 children were interviewed, and 5532 of them answered that their children were sick in the past two weeks prior to the survey. A total of 4979 women with complete information who met the above criteria were included for data analysis in this study (Figure 1).

###  Sample Design and Data Collection

 The AHS 2015 used a stratified two-stage sample design.^31^ Within each province (stratum), 30 clusters were randomly selected from the list of clusters provided by the central statistics office. Within each cluster, 23 households were selected randomly by the field teams.^31^ The field teams were trained surveyors and were recruited from provinces to minimize selection bias during data collection. Selection bias could have happened if the surveyors were not from the respective provinces. In such scenarios, the surveyors may have dropped the sampled cluster(s) due to security concerns or logistic challenges in the area.

 During the survey, two types of questionnaires were used: the household questionnaire, and the women’s and children’s questionnaire. The household questionnaire was used to identify women who were eligible to be interviewed for the questions in the women’s and children’s questionnaire. During the interview, the women who had under-5 children were asked the following questions related to acute childhood illness.

1. In the past 2 weeks, has (name of the child) had diarrhea?
2. In the past 2 weeks, has (name of the child) been ill with a fever?
3. In the past 2 weeks, has (name of the child) been ill with a cough?
4. In the past 2 weeks, has (name of the child) had fast, or short and rapid breathing, or difficulty breathing?
  If the response was “yes” for any of the above questions, the following question was asked.
5. Did you seek any advice or treatment from any source?
  If the response was “yes” the following question was asked.
6. From where you FIRST sought advice or treatment?

**Figure 2:**
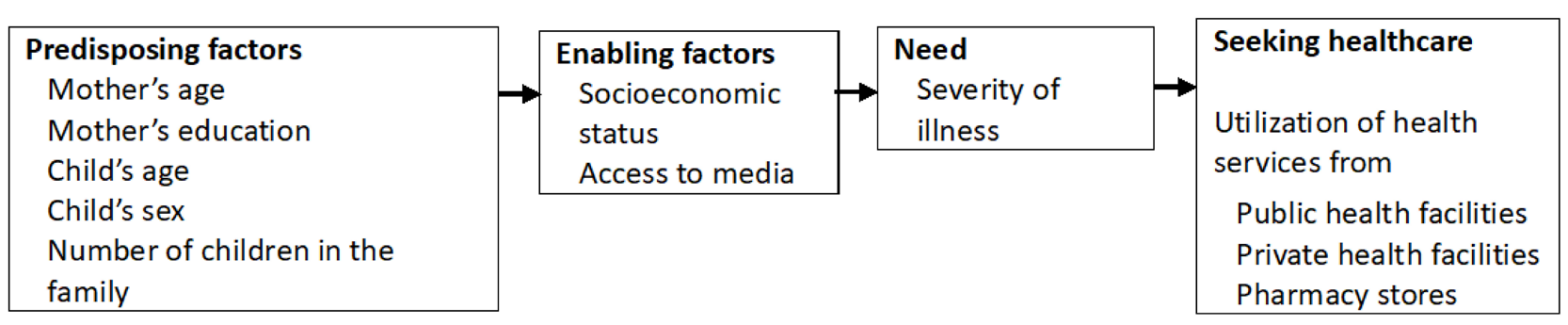
Theoretical framework for Mothers’ Healthcare Seeking Behavior for Unwell Children.

We used a modified version of the Andersen’s behavioral model for health services that was employed in previous studies,^32,33^ and created a theoretical framework for mothers’ care seeking behavior for unwell children (Figure 2).

###  Statistical Analysis

 Data from women whose under-5 children were sick with fever, diarrhea, cough, or had rapid breathing or difficulty breathing in the past 2 weeks were included and analyzed. Cases with complete information on the outcome variable and explanatory variables were included for data analysis. The outcome variable (a four-categorical variable) was created as healthcare was sought from a public clinic (category = 1), from a private clinic (category = 2), from a pharmacy store (category = 3) vs. healthcare was not sought (category = 0), using the questions “Did you seek any advice or treatment from any source?” and “From where you first sought advice or treatment?”

 The explanatory variables were mother’s age (15-29 years, 30-39 years, and 40-49 years), child’s age (<2 years, 2 to under 4 years, and 4 to under 5 years), child’s sex, mother’s education level (no formal education, completed primary education, completed secondary or higher education), socioeconomic status (quintile), number of under-5 children in the household (1 child, 2-3 children, and ≥4 children), access to radio (never listened to radio vs. listened to radio), access to TV (never watched TV vs. watched TV), and mother’s knowledge of symptoms of childhood illness (knew no symptom, knew 1 symptom, knew 2-3 symptoms, knew 4-7 symptoms). We used the mothers’ knowledge of symptoms as a measure of severity of childhood illness perceived by the mother (See Table 1). The level of perceived severity was determined by the number of symptom(s) a mother knew.

**Table 1:** Women’s Perceived Severity of Childhood Illness

**The Question on Severity of Childhood Illness**	**Response Options (Symptoms)**
What type of symptoms would cause you to take your child to a health facility right away?	1. Child cannot suckle breastmilk
	2. Child becomes sicker
	3. Child develops a fever
	4. Child has fast breathing
	5. Child has difficulty breathing
	6. Child has blood in stool
	7. Child is drinking poorly
	8. Others (specify)
Instructions to surveyor(s). Record all responses the respondent provides. Do not make any suggestion.	

 The variable on socioeconomic status was created using the principal component analysis, using the household ownership of assets, household use of amenities of life, and construction material used for the household properties. Using the “pca” function in Stata version 13, the correlation matrix between the variables of household assets, household use of amenities, and construction material used for the household properties was obtained. The first component, which explained 11% variance in the original variables related to household assets, amenities, and construction material, was used to create quintile of socioeconomic status.

 Multicollinearity between explanatory variables was examined by using the variance inflation factors (VIF)function in Stata version 13, and we found that the highest VIF value was 1.29 which was for the variable on socioeconomic status. Given the highest VIF value was 1.29, we concluded that there was no indication of substantial multicollinearity between variables.^34^

 For multivariable analysis, a generalized linear model with a multinomial outcome was specified as shown below.


$$Y_{ij}=\beta_{0}+\sum_{k=1}^{K}\beta_{j}X_{ij}+\varepsilon_{ij}$$

*Y*_ij_ refers to the outcome variable for woman *i* (whether she sought healthcare from a public clinic; whether she sought healthcare from a private clinic, whether she sought healthcare from a pharmacy store, with the response of “yes/no” for each of them), with *j* category of explanatory variables. *X*_ij_ denotes a vector of explanatory variables, and *k* refers to the number of explanatory variables. β_0_ stands for the intercept term, and the exponential of β_j_ provides the odds ratio (OR) of seeking healthcare for each category of explanatory variables. *ɛ*_ij_ refers to the error term. Provincial weights based on number of households were used. Data were analyzed in Stata version 13 using the “svyset,” “strata,” and “svy” functions before running the “mlogit” function in the multinomial logit model to account for clustering and weighting. The ORs were obtained by the “mlogit, rrr” function.

## Results

Table 2 shows that there were 4979 ever-married women, aged 15-49 years, whose under-5 children were unwell in the past 2 weeks. Thirty-nine percent of women sought healthcare from a health provider for their children who had fever, cough, faster breathing, or diarrhea. There were significant differences between the mothers who sought healthcare and those who did not seek healthcare for their children. Higher proportions of older mothers sought healthcare for their children, and for a higher proportion of children <2 years healthcare was sought. For a significantly higher proportion of boys, and for a significantly higher proportion of children whose mothers knew ≥4 symptoms of childhood illness healthcare was sought. In households with 1 child, for a substantially higher proportion of children healthcare was sought. An unusual finding was that for a higher proportion of children whose mothers watched TV, healthcare was not sought (details in Table 2). Weighted proportions for the baseline characteristics of mothers and children are provided in Supplementary file 1.

**Table 2:** Baseline Characteristics of Mothers and Their Under-5 Children

		**All women and Children** **No. (%)**	**Those Who Sought Care ** **No. (%)**	**Those Who Did not Seek Care ** **No. (R)**	***P***** Value**
Total	Women or children	4979 (100.0)	1947 (39.1)	3032 (60.9)	<.001
Age of mother (y)	15-29	2678 (54.0)	985 (50.6)	1693 (55.8)	.001
	30-39	1642 (33.0)	675 (34.7)	967 (31.9)	
	40-49	659 (13.0)	287 (14.7)	372 (12.3)	
Age of child (y)	<2	1782 (36.0)	827 (42.5)	955 (31.5)	<.001
	2-<4	2196 (44.0)	843 (43.3)	1353 (44.6)	
	4-<5	1001 (20.0)	277 (14.2)	724 (23.9)	
Gender of child	Boy	2659 (53.4)	1083 (55.6)	1576 (52.0)	.012
	Girl	2320 (46.6)	864 (44.4)	1456 (48.0)	
Education Level of women	No education	4089 (82.1)	1602 (82.3)	2487 (82.0)	.65
	Primary education	647 (13.0)	245 (12.6)	402 (13.3)	
	Secondary or higher education	243 (4.9)	100 (5.1)	143 (4.7)	
Socioeconomic status	Lowest (quintile)	1007 (20.2)	393 (20.2)	614 (20.3)	.010
	Low	994 (20.0)	412 (21.2)	582 (19.2)	
	Middle	1017 (20.4)	417 (21.4)	600 (19.8)	
	High	984 (19.8)	338 (17.4)	646 (21.3)	
	Highest (quintile)	977 (19.6)	387 (19.9)	590 (19.5)	
Perceived severity of childhood illness	Knowing no symptom	210 (4.2)	53 (2.7)	157 (5.2)	<.001
	Knowing 1 symptom	840 (16.9)	305 (15.7)	535 (17.7)	
	Knowing 2-3 symptoms	3036 (61.0)	1182 (60.7)	1854 (61.2)	
	Knowing ≥4 symptoms	893 (17.9)	407 (20.9)	486 (16.0)	
Number of under-5 children in household	1 child	2138 (42.9)	1051 (54.0)	1087 (35.8)	<.001
	2-3 children	2707 (54.4)	877 (45.0)	1830 (60.4)	
	≥ 4 children	134 (2.7)	19 (1.0)	115 (3.8)	
Women listen to radio	Yes	2739 (55.0)	1040 (53.4)	1699 (56.0)	.07
	No	2240 (45.0)	907 (46.6)	1333 (44.0)	
Women watch TV	Yes	2433 (48.9)	917 (47.1)	1516 (50.0)	.046
	No	2546 (51.1)	1030 (52.9)	1516 (50.0)	

Note: *P* values were obtained from chi-square test for cross-tabulations of characteristics between mothers who sought healthcare and mothers who did not. A two-sample test of proportions was conducted to obtain *P *value for women who sought care (39.1%) and women who did not seek care for their children (60.9%).

**Figure 3:**
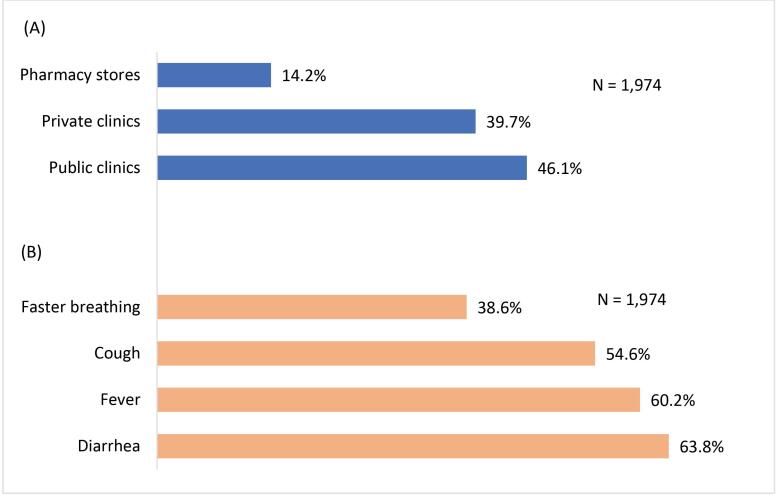
(A) Proportions of Maternal Care-Seeking for Children by Health Provider Type and (B) Proportions of Children Receiving Care by Illness Type.

Figures 3A and 3B show the proportions of mothers who sought healthcare for their children, by health provider type, and the proportions of children for whom healthcare was sought, by illness type, respectively. Public clinics were the facilities from where healthcare for children was sought by 46.1% of mothers, followed by private clinics (39.7%), and pharmacy stores (14.2%). The most common illnesses the children had, and healthcare was sought for, were diarrhea (63.8%), followed by fever (60.2%), cough (54.6%), and faster breathing (38.6%).

Table 3 shows results from multivariable analysis. Mother’s age, child’s age, child’s sex, socioeconomic status, mothers’ perceived severity of childhood illness, and number of under-5 children were predictors of mothers’ care seeking behaviour for childhood illness. It shows that child’s age is a significant predictor of mothers’ care seeking behavior for childhood illness, as the likelihood of mothers’ care seeking was significantly low for older children (Adjusted OR 0.53 [0.41-0.69] from private clinics; 0.63 [0.46-0.88] from pharmacy stores for children 2 years to under-4 years), and [Adjusted OR 0.51 [0.37-0.70] from public clinics, 0.33 [0.23-0.49] from private clinics, and 0.36 [0.22-0.61] from pharmacy stores for children 4 years to under-5 years), compared to under-2 years old. It was more likely for mothers, aged 40-49 years, to seek healthcare for children (Adjusted OR 1.54 [1.11-2.12] from private clinics) compared to mothers, aged 15-29 years (details in Table 3).

**Table 3:** Predictors of Mothers’ Care Seeking for Childhood Illness by Health Facility Type (N = 4979)

		**Public Clinics**	**Private Clinics**	**Pharmacy Stores**
		**Adjusted OR (95% CI)**	**Adjusted OR (95% CI)**	**Adjusted OR (95% CI)**
Age of mother (y)	15-29	Ref	Ref	Ref
	30-39	1.21 (0.97-1.52)	1.14 (0.88-1.48)	1.16 (0.83-1.63)
	40-49	1.03 (0.78-1.38)	1.54^b^ (1.11-2.12)	1.32 (0.81-2.13)
Age of child (y)	<2	Ref	Ref	Ref
	2 to <4	0.81 (0.64-1.01)	0.53^c^ (0.41-0.69)	0.63^b^ (0.46-0.88)
	4 to <5	0.51^c^ (0.37-0.70)	0.33^c^ (0.23-0.47)	0.36^c^ (0.22-0.61)
Gender of child	Boy	Ref	Ref	Ref
	Girl	0.95 (0.78-1.16)	0.74^b^ (0.59-0.93)	1.15 (0.85-1.56)
Education level of woman	No education	Ref	Ref	Ref
	Primary education	0.88 (0.64-1.22)	0.90 (0.62-1.31)	0.76 (0.49-1.18)
	Secondary education	1.58 (0.94-2.67)	0.94 (0.57-1.52)	1.23 (0.63-2.42)
Socioeconomic status	Lowest (quintile)	Ref	Ref	Ref
	Low	1.21 (0.94-1.57)	1.20 (0.86-1.67)	1.42 (0.87-2.32)
	Middle	1.03 (0.78-1.35)	1.20 (0.87-1.64)	1.12 (0.67-1.89)
	High	0.73 (0.53-1.01)	1.01 (0.71-1.42)	1.20 (0.70-2.04)
	Highest (quintile)	0.39^c^ (0.26-0.59)	1.72^c^ (1.24-2.40)	1.49 (0.90-2.46)
Perceived severity of childhood illness	Knows none	Ref	Ref	Ref
	Knows 1 symptom	1.67 (0.79-3.54)	1.37 (0.75-2.51)	2.01 (0.81-5.01)
	Knows 2-3 symptoms	2.29^a^ (1.13-4.65)	1.40 (0.82-2.38)	3.98^c^ (1.71-9.28)
	Knows ≥ 4 symptoms	2.97^c^ (1.44-6.16)	1.67 (0.94-2.97)	7.20^c^ (3.04-17.04)
Number of under-5 children in household	1 child	Ref	Ref	Ref
	23 children	0.53^c^ (0.43-0.65)	0.61^c^ (0.48-0.79)	0.51^c^ (0.37-0.69)
	≥4 children	0.14^c^ (0.06-0.35)	0.27^c^ (0.13-0.56)	0.29 (0.04-2.08)
Listens to radio	No	Ref	Ref	Ref
	Yes	1.05 (0.85-1.30)	1.11 (0.90-1.38)	1.11 (0.82-1.50)
Watches TV	No	Ref	Ref	Ref
	Yes	0.96 (0.76-1.22)	0.98 (0.80-1.21)	1.30 (0.91-1.84)

Abbreviations: OR, odds ratio; CI, confidence interval. ^a^*P* < .05, ^b^*P* < .01, ^c^*P* < .001.

**Figure 4:**
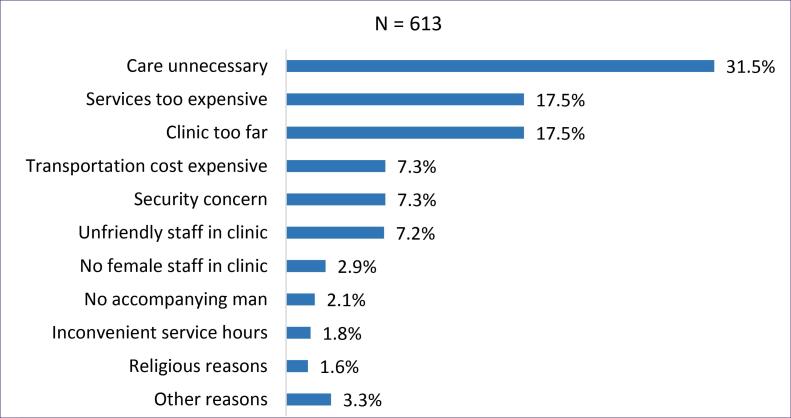
Reasons for not Seeking Healthcare for Unwell Children. Note: 2419 women (out of 3032) did not answer this question.

 Data on the reasons for not seeking healthcare were available for and analyzed from 613 out of the 3032 mothers who did not seek healthcare for their unwell children. Figure 4 shows that 31.5% of mothers thought it was unnecessary to seek healthcare, 17.5% thought services were expensive, 17.5% responded the clinic was too far, 7.3% thought transportation cost was too expensive, 7.3% expressed security concern, and 7.2% responded that staff in the clinic were unfriendly. Ranging from 1.6% to 2.9% of mothers reported the following reasons for not seeking healthcare for their unwell children: unavailability of female staff in the clinic, inconvenience service hours, religious reasons, and lack of an accompanying man.

## Discussion

 The discussion has been structured considering the Andersen’s model used for the theoretical framework in this study. This study revealed that only 39.1% of women sought healthcare for childhood illness. Previous studies from LMICs reported that 45% to 65% of mothers sought healthcare for childhood illness.^22,35-37^ With respect to predisposing factors, our study showed that child’s age is a strong predictor of mothers’ care seeking behavior for childhood illness, and our finding supports those previously reported from LMICs.^22,32,37^ We observed lower odds of healthcare seeking for girls, which is consistent with findings from several LMICs.^32,38-40^ A plausible reason could be related to the parents preference for male children by some cultures, including those in Afghanistan.^39,41^ In our study it was found that mother’s age is a strong predictor of mothers’ care seeking behavior for childhood illness. A recent study in LMICs showed that mother’s age is a potential, but not significant, predictor of mothers’ care seeking behavior for childhood illness.^35^ A plausible reason for our finding could be attributed to the experience mothers gain for seeking healthcare for their children throughout their life span. Our findings that mother’s education is not a strong predictor of healthcare seeking for sick children contradicts findings previously reported from LMICs.^17,32,35^ Despite our findings on the effect of mother’s education on healthcare seeking behavior, we cannot rule out the pivotal role of mother’s education on healthcare seeking for childhood illness because of the abondance of evidence reported from several studies.^17,32,35^ However, our finding that number of children in a household is a strong predictor of mothers’ care seeking behavior for childhood illness is consistent with those previously reported.^35^

 With respect to enabling factors, our study identified that socioeconomic status is a significant predictor of mothers’ care seeking behavior for childhood illness, and this is consistent with previous findings from LMICs.^22,32,35,42^ Our findings that odds of healthcare seeking from private clinics was higher for children from wealthier families support those previously reported.^32^ Unlike above, our findings that mothers’ exposure to radio and television did not affect mothers’ care seeking behavior for childhood illness contradict those from previous studies.^35,43^ Although, our findings contradict the significant role of mothers’ access to media on care seeking behavior, recent data from Afghanistan indicate the significant effects of women’s exposure to media on care seeking behavior.^44,45^

 With respect to perceived severity of illness, which could prompt the need to seek healthcare, our findings support those previously reported from LMICs.^22,32,33^ Unlike these studies, however, our findings provided a dose-response relationship between the number of symptoms of childhood illness that a mother knows and the likelihood of healthcare seeking for the unwell children. This means the more symptoms a mother knows the higher would be the likelihood of healthcare seeking for the unwell children. A previous study found that mothers’ awareness of childhood illness, perceived importance of early treatment, and perceived severity were significant predicators of mothers’ care seeking behavior for childhood illness.^22^ The authors defined perceived illness severity when a mother thought that her sick child was severely ill. Another study found strong associations between mothers’ knowledge of symptoms in a child and healthcare seeking for the sick child,^32^ and examined the symptoms related to diarrhoea in children who received health services for the treatment of diarrhoea, and the symptoms related to cough and fever in children who received health services for the treatment of cough and fever. In our study, we defined mothers’ perceived severity of childhood illness when a mother knew symptoms in a sick child that alerted her to take the child to a health provider right away. Symptoms such as inability of a child to suckle breastmilk, become sicker, develop a fever, have fast breathing, have difficulty in breathing, have blood in stool, or drink poorly were considered mothers’ perceived severity of childhood illness; likewise, the number of symptoms indicated the degree of illness severity. In our study, there were few mothers who provided responses which were not among the seven symptoms we used as the mothers’ perceived severity of illness. These responses were heterogenous and it was not possible to categorize them in a specific category, and they were not analysed.

 Our findings on mothers’ perceived severity of childhood illness, predictors of mothers’ care seeking behavior, and the low prevalence of healthcare seeking behavior by mothers for childhood illness have the potential to influence health policies and interventions to promote healthcare seeking behavior for childhood illness. This is particularly important for the current situation to prioritize more targeted interventions to maximize the use of limited resources available in the health sector in Afghanistan. Health interventions which accommodate activities to improve mothers’ care seeking behavior for childhood illness may lead to the rationale use of available limited resources at the primary care level. This may help reduce the utilization of more expensive therapeutic interventions at the secondary care level.

 The simple question on mothers’ knowledge of symptoms of childhood illness with related response options in our study can be used to target health education activities related to common childhood illnesses. Research shows that early healthcare seeking for childhood illness can affect the use of health services offered from health facilities at the primary care level versus at the secondary care level.^37^ Our findings showed that one third of women, who did not seek healthcare for their unwell children, thought that care was not necessary. This emphasizes the need to focus more on educating women on symptoms that prompt healthcare seeking for unwell children. This should be noted that these findings are from data collected in 2015, which means that the extent of the reasons for not seeking healthcare may have changed over time, especially after the take-over of political power by the Taliban in August 2021. Under the Taliban’s rule, more women may not seek healthcare for their children because of the higher cost of transportation, services, and because of the shortage of essential drugs and health staff, even though security concern may have decreased in most parts of Afghanistan.

 The strengths of our study are the use of data from a nationally representative database, and the use of questionnaires and having a study design similar to those used in the demographic health surveys.^35^ This allows generalization of our findings at the national level, and the comparison of our findings with findings from other LMICs. The sample size is sufficiently large, and this can increase the validity of our findings.

 The limitations of our study are the chance of social desirability and recall bias,^46^ though the change of recall bias may have been minimal because the interviewee mother was asked about the events for her child in the past 2 weeks prior to the survey. Another limitation is the lack of data on access to health facilities. Research shows that there is a strong association between distance to health providers and healthcare seeking behavior for childhood illness.^40,43,47^ A further limitation of our study is that the variable used to measure socioeconomic status did not capture it very well.

 Future research may examine the association between distance to health facilities and healthcare seeking behavior, including delays in seeking healthcare and use of health services from primary care facilities versus hospitals. Another venue to explore may be the cost of using health services, including transportation cost and its association with healthcare seeking for childhood illness.

## Conclusion

 Effective health policy interventions are needed to enhance mothers’ care seeking behavior for childhood illness. Our findings reinforce the need for targeted interventions to enhance mothers’ care seeking behavior for common childhood illnesses. The targeted interventions should focus on improving mothers’ perceived severity of childhood illness through increasing their knowledge of symptoms that would prompt actions to take the unwell child to a health provider. The interventions should focus to encourage parents to seek healthcare for their under-5 children, particularly those <2 years old, regardless of the child’s sex and number of children living in the household.

## Acknowledgements

 We thank the Silk Route Training and Research Organization (SRTRO) for sharing the data the organization collected for the AHS 2015.

## Ethical issues

 Secondary data were used, and it was not required to obtain an ethics approval. The data used in this study did not include identifiable personal information.

## Competing interests

 Authors declare that they have no competing interests.

## Supplementary files

Supplementary file 1. Baseline Characteristics of Women and Their Children.
