# Supplementary material for: Predictors of Mothers’ Care Seeking Behavior for Common Childhood Illnesses: Findings From the Afghanistan Health Survey 2015
Source: Int J Health Policy Manag. 2023 Nov 26;12:7598. doi: 10.34172/ijhpm.2023.7598 (PMC10843312; doi:10.34172/ijhpm.2023.7598)
Supplement: Supplementary file 1 — Baseline Characteristics of Women and Their Children. [file ijhpm-12-7598-s001.pdf]

Article title: Predictors of Mothers' Care Seeking Behavior for Common Childhood Illnesses: Findings From the Afghanistan Health Survey 2015

Journal name: International Journal of Health Policy and Management (IJHPM)

Authors' information: Essa Tawfiq[1]*, Khwaja Mir Islam Saeed[2,3], Sayed Ali Shah Alawi[4], Jammalluddin Jawaid[5], Syed Nasir Hashimi[6]

[1]The Kirby Institute, University of New South Wales, Sydney, NSW, Australia.

[2]Global Health Development/Eastern Mediterranean Public Health Network (GHD/EMPHNET), Amman, Jordan.

[3]Afghanistan Field Epidemiology Training Program, Afghanistan National Public Health Institute, Kabul, Afghanistan.

[4]Afghanistan Pediatric Association, Kabul, Afghanistan.

[5]Agency for Assistance and Development of Afghanistan, Kabul, Afghanistan.

[6]Jhpiego, Kabul, Afghanistan.

***Correspondence to:** Essa Tawfiq; Email: e.tawfiq@unsw.edu.au

Citation: Tawfiq E, Saeed KMI, Alawi SAS, Jawaid J, Hashimi SN. Predictors of mothers' care seeking behavior for common childhood illnesses: findings from the Afghanistan health survey 2015. Int J Health Policy Manag. 2023;12:7598. doi:10.34172/ijhpm.2023.7598

Supplementary file 1. Baseline Characteristics of Women and Their Children

	Women who did not seek care n=3032		*Women who sought care n=1,947	All women or children n=4979	
	%	(95%CI)	%	%	(95%CI)
Mother's age					
15-29 years	57.3	(54.9-59.7)	53.0	55.7	(53.8-57.6)
30-39 years	32.7	(30.4-34.9)	34.6	33.4	(31.6-35.3)
40-49 years	10.0	(8.7-11.4)	12.0	10.8	(9.7-11.9)
Child's age					
< 2 years	33.2	(30.9-35.4)	46.0	38.3	(36.4-40.2)
2 years to < 4 years	44.9	(42.6-47.1)	42.0	43.7	(41.9-45.5)
4 years to < 5 years	22.0	(20.2-23.8)	11.8	18.0	(16.7-19.3)
Child's sex					
Boy	52.9	(50.5-55.2)	55.0	53.9	(52.1-55.7)
Girl	47.1	(44.8-49.5)	45.0	46.1	(44.3-47.9)
Mother education level					
No education	77.5	(75.3-79.7)	76.8	77.2	(75.6-78.9)
Primary education	16.4	(14.5-18.4)	14.8	15.8	(14.3-17.4)
Secondary education	6.1	(4.8-7.4)	8.0	6.9	(5.8-8.1)
Socioeconomic status					
Lowest quintile	15.8	(14.4-17,1)	15.4	15.7	(14.6-16.8)
Low	14.0	(12.7-15.2)	16.8	15.1	(14.1-16.2)

Middle	17.5	(16.0-19.1)	18.3	17.8	(16.6-19.0)
High	21.0	(19.1-22.9)	17.3	19.6	(18.1-21.1)
Highest quintile	31.7	(29.3-34.0)	31.9	31.8	(29.9-33.7)
Woman's knowledge of severity of childhood illness					
Woman knows no symptom	7.3	(5.7-8.8)	3.5	5.8	(4.6-6.9)
Woman knows 1 symptom	20.9	(18.7-23.1)	16.5	19.2	(17.5-20.9)
Woman knows 2-3 symptoms	56.8	(54.4-59.2)	58.0	57.4	(55.5-59.4)
Woman knows ≥ 4 symptoms	15.0	(13.6-16,5)	22.0	17.6	(16.3-18.9)
Number of under5 children in household					
1 child	38.1	(35.7-40.4)	55.0	44.8	(42.9-46.6)
2-3 children	58.0	(55.5-60.5)	43.6	52.4	(50.5-54.3)
≥ 4 children	3.9	(2.9-4.9)	1.2	2.8	(2.2-3.5)
Access to radio					
No	45.6	(43.3-47.9)	44.9	45.3	(43.4-47.2)
Yes	54.4	(52.1-56.7)	55.0	54.7	(52.8-56.6)
Access to TV					
No	42.4	(40.1-44.6)	41.4	42.0	(40.3-43.7)
Yes	57.6	(55.4-59.9)	59.0	58.0	(56.3-59.7)

The above proportions and 95%CI were obtained by using survey weights at provincial level.
*95%CI for proportions on characteristics of women who sought care were not obtained because of the presence of single sampling unit within strata at provincial level.
